# Characteristics and preventability of medication-related admissions for acute kidney injury and dehydration in elderly patients

**DOI:** 10.1007/s00228-024-03704-7

**Published:** 2024-06-03

**Authors:** Tristan Coppes, Ankie C. M. Hazen, Dorien L. M. Zwart, Ellen S. Koster, Teun van Gelder, Marcel L. Bouvy

**Affiliations:** 1 Division of Pharmacoepidemiology and Clinical Pharmacology, Faculty of Science, Utrecht Institute for Pharmaceutical Sciences (UIPS), Utrecht University, Utrecht, The Netherlands; 2 Department of General Practice & Nursing Science, Julius Center for Health Sciences and Primary Care, University Medical Center Utrecht, Utrecht University, Utrecht, The Netherlands; 3 Department of Clinical Pharmacy & Toxicology, Leiden University Medical Centre, Leiden, The Netherlands

**Keywords:** Acute kidney injury, Dehydration, Impaired renal function, Medication-related admission, Preventability, Sick day rules

## Abstract

**Purpose:**

Patients with impaired renal function using medication that affects glomerular filtration rate are at increased risk of developing acute kidney injury (AKI) leading to hospital admissions. The risk increases during periods of dehydration due to diarrhoea, vomiting or fever (so-called “sick days”), or high environmental temperatures (heat wave). This study aims to gain insight into the characteristics and preventability of medication-related admissions for AKI and dehydration in elderly patients.

**Methods:**

Retrospective case series study in patients aged ≥ 65 years with admission for acute kidney injury, dehydration or electrolyte imbalance related to dehydration that was defined as medication-related. General practitioner’s (GP) patient records including medication history and hospital discharge letters were available. For each admission, patient and admission characteristics were collected to review the patient journey. A case-by-case assessment of preventability of hospital admissions was performed.

**Results:**

In total, 75 admissions were included. Most prevalent comorbidities were hypertension, diabetes, and known impaired renal function. Diuretics and RAS-inhibitors were the most prevalent medication combination. Eighty percent of patients experienced non-acute onset of symptoms and 60% had contacted their GP within 2 weeks prior to admission. Around 40% (*n* = 29) of admissions were considered potentially preventable if pharmacotherapy had been timely and adequately adjusted.

**Conclusion:**

A substantial proportion of patients admitted with AKI or dehydration experience non-acute onset of symptoms and had contacted their GP within 2 weeks prior to admission. Timely adjusting of medication in these patients could have potentially prevented a considerable number of admissions.

## Introduction

Acute kidney injury (AKI) is an important cause of hospital admissions and increases the risk of chronic kidney disease and death [1–3]. The risk of developing AKI is increased in elderly patients, especially with comorbid conditions like diabetes, heart failure, hypertension and/or pre-existing impaired renal function [2, 4]. These elderly patients often use medication that reduces blood pressure, circulating volume or renal blood flow, such as angiotensin-converting-enzyme inhibitors (ACE-I), angiotensin receptor blockers (ARBs), diuretics, non-steroidal anti-inflammatory drugs (NSAIDs) and sodium-glucose cotransporter-2 (SGLT-2) inhibitors. These commonly used medications become potentially hazardous, with increasing risk of developing AKI and subsequent admission [5–7], during periods where there is an additional risk of developing hypovolemia, e.g. when a patient is dehydrated because of diarrhoea, vomiting or fever (so-called “sick days”) or during heatwaves. These adverse events can potentially be prevented by temporary adjustment or discontinuation of medication at risk [8].

However, in daily clinical practice, these adverse events are easily missed or not adequately acted upon. A previous study showed that in general practice in 91% of episodes with a risk of dehydration, neither an advice to adjust high-risk medication was offered to a patient nor a referral to the hospital was made [9]. This may be partially explained by the limited knowledge and awareness of primary care providers about the harmful medication effects that can occur when a patient is at risk of dehydration [10, 11]. Despite the limited clinical evidence of the effectiveness of sick day medication guidance on patient outcomes [12, 13], there is consensus among clinical guideline developers that temporary adjustment of high-risk medication should be considered during periods with a risk of dehydration [14–16].

To be able to develop a feasible and applicable (primary care based) intervention to prevent hospital admissions due to AKI or dehydration, more knowledge is needed about which patients are at the highest risk of such admissions and whether opportunities to intervene prior to admission have been missed.

Therefore, the aim of this study is to gain insight into the characteristics and preventability of medication-related admissions for acute kidney injury and dehydration by retrospectively reconstructing the pre-admission patient journey in a cohort of elderly patients, who had been admitted to the hospital with AKI, dehydration or electrolyte imbalance related to dehydration.

## Methods

### Study design and setting

This was a retrospective case series study. A database with medication-related admissions of elderly polypharmacy patients (in total 11,928 patients, defined as being 65 years or older and using 5 or more chronic medications), recruited from 25 general practices in The Netherlands covering the period 2013–2015 was used. This database was developed as part of a previous study [17, 18] consisting of general practice data including medication history, lab results, International Classification of Primary Care (ICPC) codes and hospital data including hospital discharge letters. Medication-relatedness of admissions was assessed through a multi-step approach with physicians and clinical pharmacists including solo assessment and discussion meetings. Every medication-related admission was coded with the reason for admission, a total of 1536 medication-related admissions were identified in the previous study. For the current study, all medication-related admissions with AKI or dehydration as the primary reason for admission were included. In addition, admissions with electrolyte imbalance caused by dehydration, fever, diarrhoea or vomiting were also included.

### Data collection

For all participants, the GPs electronic medical records of the patient including medication history and laboratory results within 2 years before admission were available. ICPC codes for GP visits were available as far back as when the patient was in care of the GP. Also, for all participants, the hospital discharge letters were available. The following data were extracted: age, gender, living at home or in a nursing home, the use of potential harmful drugs (diuretics, ACE-I, ARBs, NSAIDs and metformin (SGLT2 inhibitors were not available at the time of data collection)), relevant chronic comorbidities (impaired renal function (defined as < 60 ml/min/1.73^2^ m), hypertension, diabetes, heart failure [19]), trend in renal function in the year prior to admission, renal function during admission, number and reason of GP consultations in the 2 weeks prior to admission, presence of sick days and acuteness of symptoms prior to admission.

### Preventability assessment

Preventability in this study was defined as a hospital admission that could have been prevented if one or more potentially harmful medication(s) (diuretic, ACE-I, ARB, NSAID, metformin) had been timely and adequately adjusted. Answers were recorded as yes, no or unable to assess (if too little information was available to make a well-informed decision). A case-by-case assessment of preventability of each admission was carried out. The first ten admissions were individually reviewed by a researcher and pharmacist (TC), a prescribing pharmacist in primary care (AH) and a senior clinical pharmacist (MB). Hereafter, a meeting was held to compare findings and to validate whether the assessment of preventability was clear and feasible with the available data sources. After the first meeting, two researchers (TC, AH) continued the individual assessment of the remaining admissions. Results were again compared and consensus was reached. For the admissions where consensus could not be reached, an expert (GP specialised in geriatrics) was consulted to make the final decision.

### Analysis

Descriptive analysis of the patient characteristics, the preventability and distribution of a combination of comorbidities and medications in use were performed using IBM SPSS Statistics for Windows Version 27.0. Continuous variables were reported as a mean with standard deviation (SD). Skewed distributed variables were reported as a median with an interquartile range (IQR).

## Results

In the previous study [18], 1536 possible medication-related hospital admissions were identified. In this study, 75 of these admissions were selected with their primary reason for admission being AKI, dehydration or electrolyte imbalance related to dehydration.

### Patient characteristics

The median age of patients was 81 years and 53% were female. The median eGFR at the time of hospital admission was 21 ml/min/1.73 m^2^. A total of 80% of patients had hypertension, 51% diabetes mellitus, 36% heart failure and 57% impaired renal function. All patients used at least one drug that could compromise the glomerular filtration rate, most commonly a diuretic (90%). The majority (80%) used a combination of 2–4 different potentially harmful drugs (Table 1).

**Table 1:** Patient characteristics of all admissions (*n* = 75)

**Variable**	**All admissions (*n*** **= 75)**^a^
Female sex, ***n*** (%)	40 (53)
Age, median (IQR)	81 (74–86)
eGFR (ml/min) at hospital admission, median (IQR)	21 (14–37)
Most recent eGFR (ml/min) prior to admission, median (IQR)	43 (29–55)
Number of comorbidities, median (IQR)	4 (4–6)
Hypertension, ***n*** (%)	60 (80)
Diabetes, ***n*** (%)	38 (51)
Heart failure, ***n*** (%)	27 (36)
Impaired renal function, ***n*** (%)	43 (57)
Chronic medications, median (IQR)	9 (6–11)
Use of at least one potentially harmful drug during sick days^b^, ***n*** (%)	75 (100)
Use of more than one potentially harmful drug, ***n*** (%)	60 (80)
Diuretic, ***n*** (%)	68 (90)
Loop diuretic, ***n*** (%)	49 (65)
Thiazide, ***n*** (%)	21 (28)
Potassium-sparing diuretic, ***n*** (%)	20 (27)
ACE-I, ***n*** (%)	36 (48)
ARB, ***n*** (%)	17 (23)
NSAID, ***n*** (%)	9 (12)
Metformin, ***n*** (%)	18 (24)
Use of multidose drug dispensing system, ***n*** (%)	26 (35)
Living in a nursing home or care home, ***n*** (%)	5 (7)^c^

*IQR* interquartile range, *eGFR* estimated glomerular filtration rate, *ACE-I* angiotensin converting enzyme inhibitor, *ARB* angiotensin receptor blocker

^a^A total of 75 admissions have been included from 72 different patients. Three patients had a second admission during the study period

^b^SGLT2 inhibitors were not yet included in diabetes and heart failure guidelines during the time of data collection of admissions (2013–2015)

^c^Missing data for 17 patients

### Combinations of comorbidities and drugs

As shown in Table 2, hypertension with diabetes is the most common combination of comorbidities (34) followed by impaired renal function with hypertension [25]. Thirty-three (44%) patients had a combination of more than 2 chronic comorbidities.

**Table 2:** Most prevalent combinations of comorbidities in included admissions (*n* = 75)

	**Impaired renal function** (*n* = 43)	**Hypertension** (*n* = 60)	**Heart failure** (*n* = 27)	**Diabetes** (*n* = 38)
**Impaired renal function** (*n* = 43)	4^a^	25	14	24
**Hypertension** (*n* = 60)		7^a^	22	34
**Heart failure** (*n* = 27)			3^a^	15
**Diabetes** (*n* = 38)				1^a^

^a^A combination of the same comorbidity indicates that only this comorbidity was present

As shown in Table 3, the use of a loop diuretic with a RAS-inhibitor (33) was the most common drug combination. Seventeen (23%) patients used more than 2 potential harmful drugs at time of admission.

**Table 3:** Most prevalent combination of drugs in included admissions (*n* = 75)

	**Loop diuretic** (*n* = 49)	**Thiazide diuretic** (*n* = 21)	**Potassium sparing diuretic** (*n* = 15)	**RAS- inhibitor** (*n* = 53)	**NSAID** (*n* = 9)	**Metformin** (*n* = 18)
**Loop diuretic** (*n* = 49)	8^a^	2	18	33	4	12
**Thiazide diuretic** (*n* = 21)		3^a^	1	17	1	5
**Potassium sparing diuretic** (*n* = 15)			0^a^	12	2	6
**RAS-inhibitor** (*n* = 53)				3^a^	4	17
**NSAID** (*n* = 9)					2^a^	1
**Metformin** (*n* = 18)						0^a^

^a^A combination of the same drug class indicates that only this drug class was present

*RAS-inhibitor* renin-angiotensin system inhibitor

**Table 4:** Characteristics related to the included admissions (*n* = 75)

Variable	All admissions (*n* = 75)
Trend in renal function^a^	
Stable, ***n*** (%)	26 (35)
Fluctuating, ***n*** (%)	28 (37)
Insufficient data, ***n*** (%)	21 (28)
Consult with GP within the last 14 days prior to admission, ***n*** (%)	45 (60)
Concerning “sick day symptom” prior to admission, ***n*** (%)	14 (31)
Reduced intake prior to admission, ***n*** (%)	35 (47)
Clinical picture prior to admission^b^	
Acutely unwell, ***n*** (%)	10 (13)
Not acute but gradually deteriorating over time, ***n*** (%)	60 (80)
Asymptomatic, ***n*** (%)	5 (7)
Died during or soon after admission, n (%)	5 (7)
Any hospital admission 1 year prior to index admission, n (%)	28 (37)

^a^Fluctuating renal function was defined as a change of at least 15% in eGFR compared to the previous measurement

^b^The clinical picture prior to admission was determined through the hospital discharge letter of the admission in addition to the GP visit data if available

### Sick day and admission characteristics

More than one-third of the patients (37%) had fluctuating rather than stable renal function (35%) prior to admission. Limited intake of fluids and/or food was reported in 47% of the admissions. A minority of patients (13%) had an acute onset of symptoms on the day of admission. Less than half (37%) had a hospital admission within the year prior to the index admission. More than half of the patients (60%) had consulted the GP within the last 14 days prior to admission, of which 31% related to a “sick day symptom” like fever, diarrhoea or vomiting. There were various other reasons, not necessarily related to the reason for hospital admission, why patients contacted the GP in the 14 days prior to admission, ranging from anxiety to oedema to general malaise. Five patients died during or soon after the admission (Table 4).

### Preventability assessment

From the 72 admissions assessed, 29 (40%) were considered potentially preventable if a high-risk medication (diuretic, RAS-I, NSAID, metformin) had been timely and adequately adjusted. For three admissions, preventability was not assessed because of insufficient data.

## Discussion

This study showed that in elderly patients with a medication-related admission with the primary reason for admission being AKI, dehydration or electrolyte imbalance related to dehydration, only 13% of the patients presented with acute onset of symptoms on the day of admission. In 60% of the admissions, there had been a GP visit within 2 weeks prior to admission. Approximately 40% of the admissions were considered potentially preventable if high-risk medication (diuretic, ACE-I, ARB, NSAID or metformin) had been timely and adequately adjusted.

This study thoroughly describes the pre-admission characteristics of admitted patients due to AKI, dehydration or electrolyte imbalance related to dehydration. More than half of these patients had impaired renal function and diabetes. In addition, around 80% had hypertension and used more than 1 high-risk drug. This was also found by Duong et al. who described that hypertension was present in 72% of the patients and 78% used at least 1 high-risk drug [20] before being admitted to the hospital with community-acquired AKI. Although it would be interesting to compare the characteristics of the patients in this study with those in a control group, it is difficult to determine the ideal control group. Whether it’s healthy individuals or patients admitted for other reasons. For now, providing a detailed depiction of these patients seems to offer ample insights for clinical application. In clinical practice, the abovementioned characteristics of patients are often well-known or easy to identify by the GP or pharmacist. To prevent admissions due to AKI, creating awareness in the primary care team by, for example, highlighting high-risk patients in the patient medical file could be an important first step. By doing this, high-risk patients who contact a healthcare professional with sick day symptoms might be timely detected and receive adequate information, recommendations and warnings because the pharmacy technician or general practice nurse recognizes that it concerns a high-risk patient. To make sure the group of patients does not exceed a manageable scale, characteristics like age, chronic kidney disease and the use of multiple high-risk drugs should be combined to make sure that patients with the highest risk receive a label.

Another important factor to be aware of is whether the patient had a previous hospital admission. Our study showed that in almost 40% of the admissions, patients were admitted to the hospital within 1 year prior to the index admission. This is in line with previous studies showing an increased risk of hospital admission in patients with a previous admission [21, 22]. In addition, 60% of patients had already visited the GP within 14 days of admission. GPs and pharmacists should be more alert to the combination of symptoms, comorbidities, medication, factors of frailty and the potential risk of dehydration during sick days for individual patients. For example, during GP visits there should be extra attention to certain symptoms, like loss of appetite, fever or nausea. If these symptoms occur, extra focus should be on patient education and temporary adjustment of high-risk medication should be considered to prevent dehydration. On the other hand, there are also potential risks of discontinuing medication that GPs and patients should be aware of. Potential risks could be, for example, that patients do not adequately restart their medication after temporary discontinuation, or heart failure patients who risk decompensating without their diuretics. Therefore, clear instructions for patients and education for healthcare professionals about patient-specific monitoring, i.e. monitoring weight in heart failure patients, is paramount. Although, for the majority of patients a short discontinuation of fluid-reducing medication, in a situation where the fluid intake and/or uptake is lowered too (during a sick day), should not pose a direct threat to their health status.

The admission characteristics showed that only 16% of patients presented with an acute onset of symptoms. This shows that there is a potential window of opportunity for healthcare providers like GPs, homecare nurses or pharmacists, for an intervention. However, in some admissions, it only became clear at the emergency department of the hospital that the patient had gradually developed symptoms related to AKI or dehydration. This emphasizes the need to focus on educating high-risk patients, whose knowledge about the topic is poor [23], and their carers about the significance of notifying a healthcare provider when a patient develops symptoms, even a minor illness [12, 24]. In addition, patient counselling should also involve educating about the risk of dehydration during heatwaves with lifestyle and personalised medication recommendations. Information materials, for example, the Scottish Sick Day Rules card [25], should be provided during prescribing and dispensing at the pharmacy. There are also opportunities for homecare nurses to educate the patient during heatwaves and to refer to the GP or pharmacist, if necessary.

However, the implementation strategies should be tailored to the characteristics of the health system of a country. Additionally, clinical practice guidelines differ in their medication-specific recommendations during sick days. For example, the guideline of the Dutch Kidney Foundation recommends temporarily halving the dose of RAS inhibitors, whereas the Scottish guideline recommends temporary discontinuation. Healthcare professionals need to make agreements about this beforehand to harmonize information provision to patients.

In this study, the preventability assessment of admissions focused on a selection of harmful medications in a restricted population. Similar results were found in Uitvlugt et al. [22] where they assessed preventability in medication-related readmissions and found a preventability of 40%. Their assessment was not limited to AKI and dehydration but included multiple hospital wards. They also found that diuretics were one of the most common causes of preventable admissions.

### Strengths and limitations

A strength of this study is that multiple data sources were used for each included admission providing us with a comprehensive pre-admission picture. The hospital discharge letter data were verified and enriched by GP data. This longitudinal information provided insight into the route of the patient from home to hospital which is crucial for studying preventability of admissions. Secondly, this is the first study that, besides looking into the characteristics, also studied the preventability of medication-related admissions due to AKI and dehydration.

This study also has limitations. Firstly, the GP data was limited to ICPC codes, medication history and laboratory results. There was no access to GP’s or practice nurse’s consultation notes. As a result, the clinical reasoning of a GP for a certain decision was not known. For example, maybe the GP intentionally decided to watch and wait rather than adjust the medication. This could have resulted in a slight overestimation of preventability of the admissions. Secondly, the preventability of the admissions was assessed with the data that was available and through expert opinion without a predefined framework or tool. This was for the current situation the most well-informed assessment available but we acknowledge that determining preventability remains a challenge and the results should be interpreted with caution.

### Future research

To reduce the number of medication-related admissions for AKI or dehydration, focussing on early detection of patients who are potentially at risk is promising. Research should focus on the possibilities of patient empowerment including recognizing risky situations themselves but also on the role that different healthcare providers can take. For example, the role of the informal caregivers and homecare nurses in signalling towards a GP when a patient is becoming ill, and also patient counselling during dispensing of medication by community pharmacists. Or pharmacotherapy optimisation by general practice pharmacists. This way, an early intervention can be performed to reduce the likelihood of a hospital admission.

Excess mortality during heatwaves is a growing problem [26, 27] and is increasing with more frequent heat waves [28]. Also, the number of home-dwelling elderly with multiple chronic diseases and medications is increasing, including the high-risk SGLT2 inhibitors [29, 30]. Future research should also include investigating potential solutions or interventions to prevent further excess of heat-related admissions and mortality.

## Conclusion

In conclusion, patients with medication-related admissions for AKI, dehydration or electrolyte imbalance related to dehydration often have multiple comorbidities and use more than one high-risk medication. Before admission, the clinical situation of patients often gradually deteriorates over the course of days or weeks and some patients visit their GP. During this time, there is a window of opportunity for a timely intervention to reduce the likelihood of admission. In this study, 40% of admissions were considered potentially preventable if high-risk medication had been timely and adequately adjusted.

## Data Availability

The data underlying this article will be shared on reasonable request to the corresponding author.
